# Minimally Invasive Resection of a Gangliocytic Paraganglioma of the Cauda Equina: A Case Report and Review of Literature

**DOI:** 10.7759/cureus.26803

**Published:** 2022-07-13

**Authors:** Nikolay Konovalov, Stanislav Kaprovoy, Muhammed Shushaev, Vasily Korolishin, Svetlana Shugay, Evgeny Brinyuk, Bakhromkhon Zakirov, Ivan Stepanov

**Affiliations:** 1 Department of Spinal and Peripheral Nerve Surgery, Burdenko Neurosurgical Center, Moscow, RUS; 2 Department of Neuropathology, Burdenko Neurosurgical Center, Moscow, RUS; 3 Department of General Surgery, Irkutsk State Medical University, Irkutsk, RUS

**Keywords:** cauda equina, miss, tubular retractors, minimally invasive spine surgery, gangliocytic paraganglioma

## Abstract

Gangliocytic paraganglioma (GP) is considered a rare neuroendocrine tumor (NET) most often located in the distal half of the duodenum. Insufficient reports describe tumors of this histological type located in the distal parts of the spinal canal, the conus medullaris and cauda equina. To date, nine cases of GP of the cauda equina and one case of GP of conus medullaris have been described. After analyzing all available treatment reports of GP, a study described it as a tumor with an extremely good prognosis in cases of total tumor removal. Here, we present a case of a female patient with a GP at the level of the L4 vertebra treated at Burdenko Neurosurgical Center using a minimally invasive approach through a tubular retractor. The tumor was removed en bloc through an intralaminar opening, and the patient was discharged two days after surgery with total regression of symptoms.

## Introduction

Gangliocytic paraganglioma (GP) is considered a rare neuroendocrine tumor (NET) consisting of three main components: epithelioid, ganglion-like, and spindle-shaped (Schwann-like) cells [1].

GP was first described as “ganglioneuroma” by Dahl et al. in 1957, and Kepes et al. coined the term “gangliocytic paraganglioma” in 1971 [2]. Despite the fact that GP is often localized in the distal half of the duodenum, there are rare reports that this tumor can also be found in the distal parts of the spinal canal (conus medullaris and cauda equina) [1,3-11]. The first report on GP of the cauda equina by Lerman et al. was published in 1972 [1]. To date, according to available literature, nine cases of GP of the cauda equina [1,3-9,11] and one case of GP of conus medullaris [10] have been described.

In this article, we describe the first case of resection of a gangliocytic paraganglioma in a 55-year-old patient using a minimally invasive spine surgery (MISS) through a tubular retractor.

## Case presentation

A 55-year-old female was referred to the Burdenko Neurosurgical Center by her primary care physician with complaints of severe lumbar pain radiating to both lower extremities along the lateral surface of the thigh and shin. About a week before admission, the patient noticed an increase in urination frequency and sensation of incomplete emptying of the bladder. On neurologic examination, no decrease in lower limb muscle strength or sensory disturbances were found. From the patient’s history, it became known that she experienced pain for about 1.5 years, for which she was treated conservatively with moderate to minimal improvement. Gradually, the symptoms progressed, and conservative pain management became ineffective.

**Figure 1:**
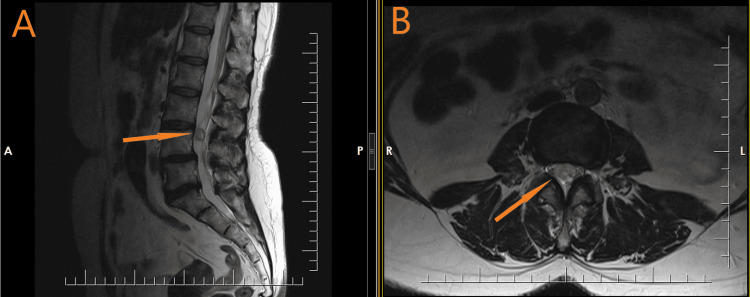
Sagittal (A) and axial (B) T2-weighted MRI showing an intradural mass at the level of the L4 vertebrae (arrows). MRI: magnetic resonance imaging

**Figure 2:**
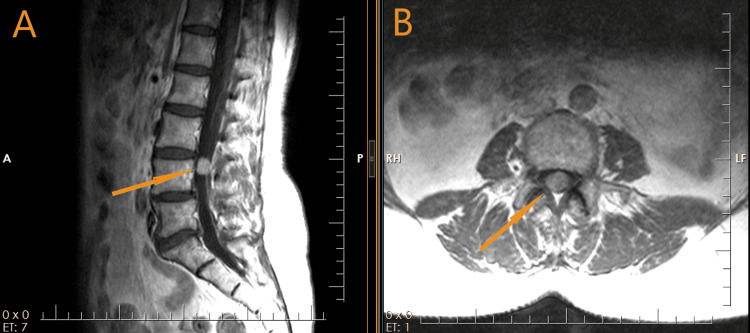
Sagittal (A) and axial (B) T1-weighted MRI with contrast enhancement showing an intradural homogeneous contrast-enhancing mass at the level of the L4 vertebrae (arrows). MRI: magnetic resonance imaging

The patient’s magnetic resonance imaging (MRI) scan of the lumbar spine revealed an intradural tumor at the level of the L4 vertebra, hyperintense in the T1-weighted image, hypointense in the T2-weighted image, and actively accumulating contrast, 1.35 × 1 cm in size (Figures 1, 2). She had no previous surgical history, and her comorbidities included hypothyroidism, for which she received treatment.

It is worth noting that the issue of preoperative verification of the histological type of tumor is difficult, and we all agree that the diagnosis becomes clear and is typically made after surgical excision [1-11]. In this connection, the preoperative diagnosis was made without specification: an intradural neoplasm at the level of the L4 vertebra.

Due to the small size of the tumor and the modern availability of low-trauma surgical techniques, we decided to excise the tumor through a minimally invasive spine surgery (MISS) using a tubular retractor system.

**Figure 3:**
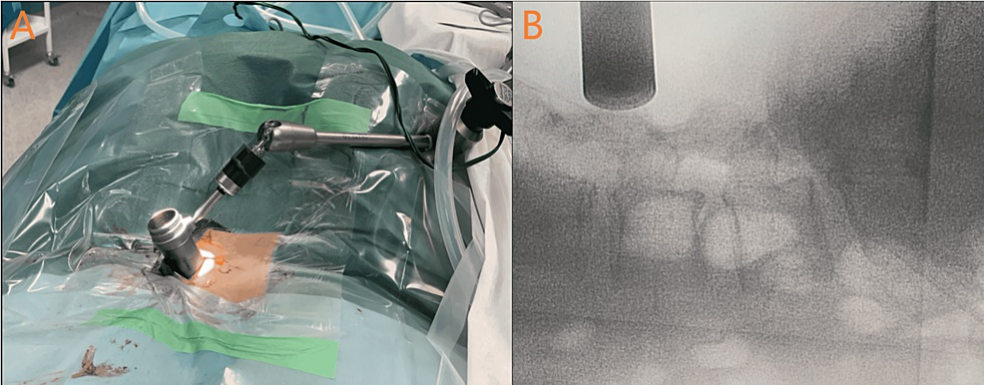
Retractor placement (A) and fluoroscopic placement control (B).

Surgery was performed the next day after admission. The patient was positioned prone on a Wilson frame, and the L4 level was identified on lateral fluoroscopy. A 2-cm skin incision 3 cm off midline was made, after which a transmuscular approach was performed using sequentially placed dilators. We used an EasyGo tubular retractor, which was inserted after the dilators, its position verified using lateral fluoroscopy and fixed to the operative table (Figure 3). Using a high-speed diamond burr, an intralaminar opening was made at the level of the L3-L4 vertebrae. The dura was opened with a linear incision, tucked, and sutured to the sides.

**Figure 4:**
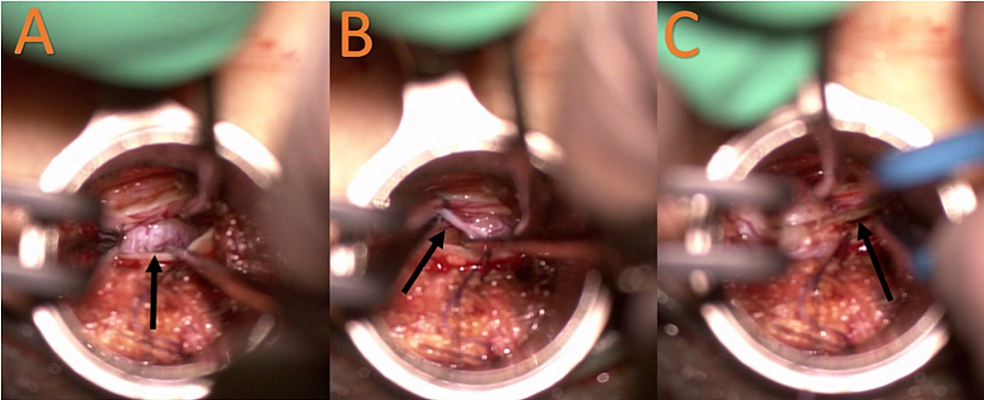
(A) Dense oval mass between the nerve roots of the cauda equina (arrow). (B) Proximal nerve root from which the tumor was growing (arrow). (C) Distal nerve root from which the tumor was growing coagulated (arrow).

Intradurally, the roots of the cauda equina were located around a dense roundish mass at the time of surgery, presumably a schwannoma. The roots of the cauda equina were moved away from the tumor, and the proximal and distal parts of the nerve from which the tumor was growing were determined. Direct stimulation of the pathological nerve root yielded no neurophysiological response, after which the distal and proximal parts of the root were coagulated and cut with microscissors (Figure 4).

**Figure 5:**
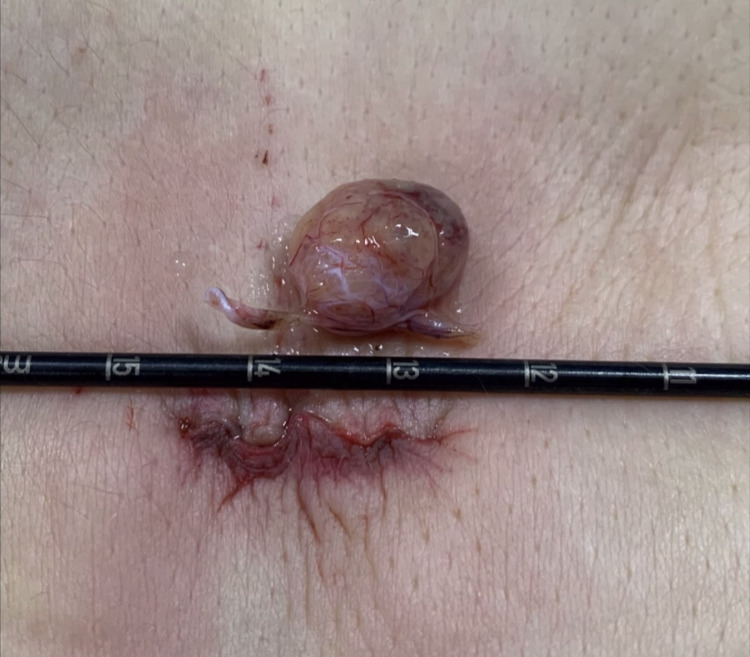
Size of the tumor in comparison with the size of the skin incision.

The tumor was removed en bloc, followed by a water-tight dural closure with 5-0 Prolene sutures and strengthened by placement of a fibrin-collagen patch. Fascia and soft tissue were sutured in a continuous manner. Figure 5 shows the size of the tumor in comparison with the size of the skin incision.

**Figure 6:**
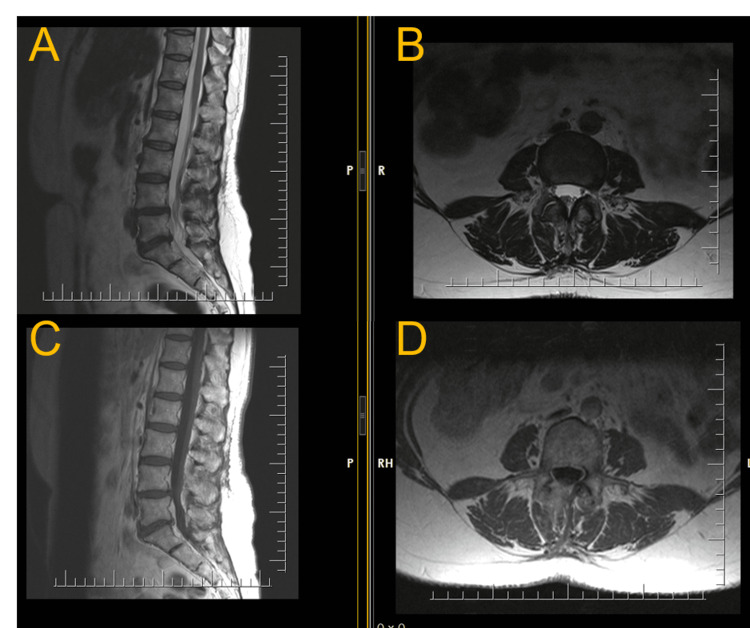
Postoperative MRI three months after surgery. Sagittal (A) and axial (B) T2-weighted images and sagittal (C) and axial (D) T1-weighted images showing no signs of the intradural mass at the level of the L4 vertebrae.

Overall, the surgery was uneventful and lasted 180 minutes. Postoperatively, the patient’s symptoms improved. The patient was discharged two days after the surgery. At discharge, the patient complained only of minimal pain in the postoperative wound, with no lower back pain or radicular pain. Bladder dysfunction regressed completely a month after discharge. Three months after surgery, a control MRI was performed (Figure 6).

**Figure 7:**
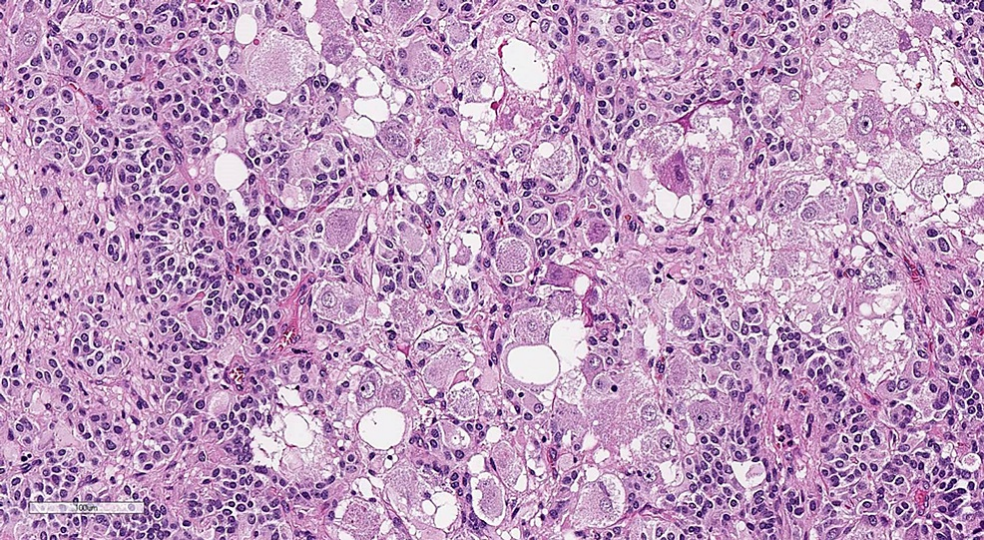
Staining with hematoxylin and eosin (×200): clusters of large ganglion-like cells surrounded by smaller chief cells.

**Figure 8:**
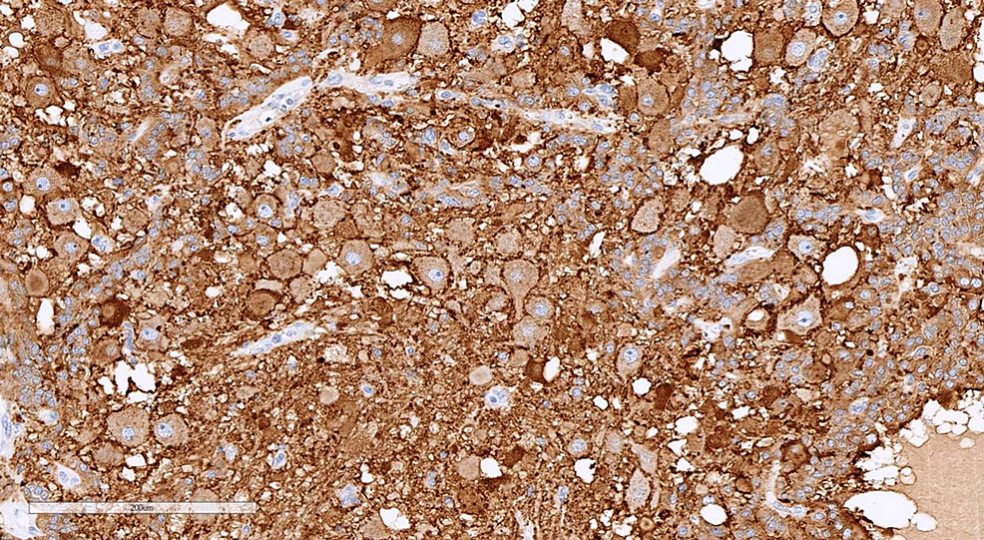
Immunohistochemical staining with synaptophysin (×200): positive expression in ganglion-like and chief cells.

**Figure 9:**
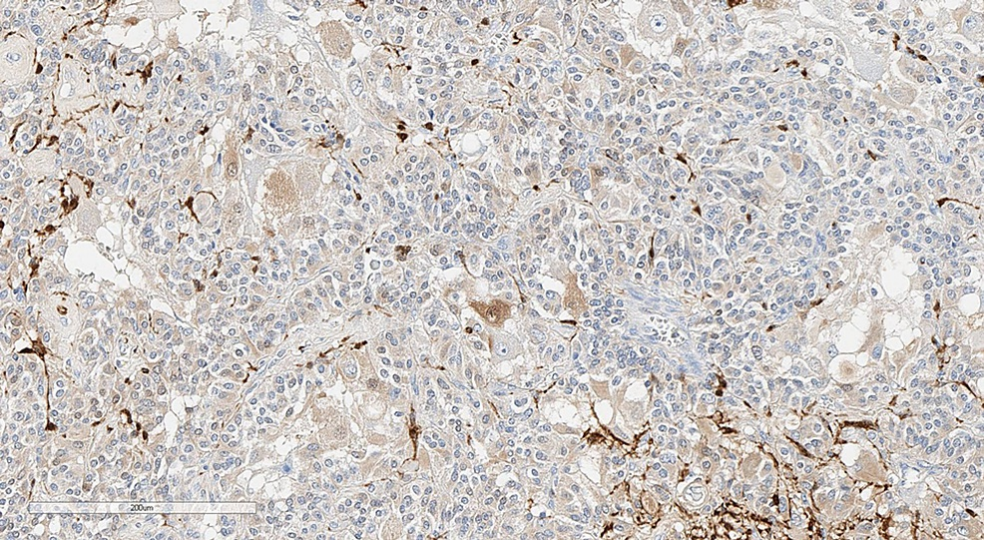
Immunohistochemical staining with S100 (×200): positive expression in a few supporting cells.

**Figure 10:**
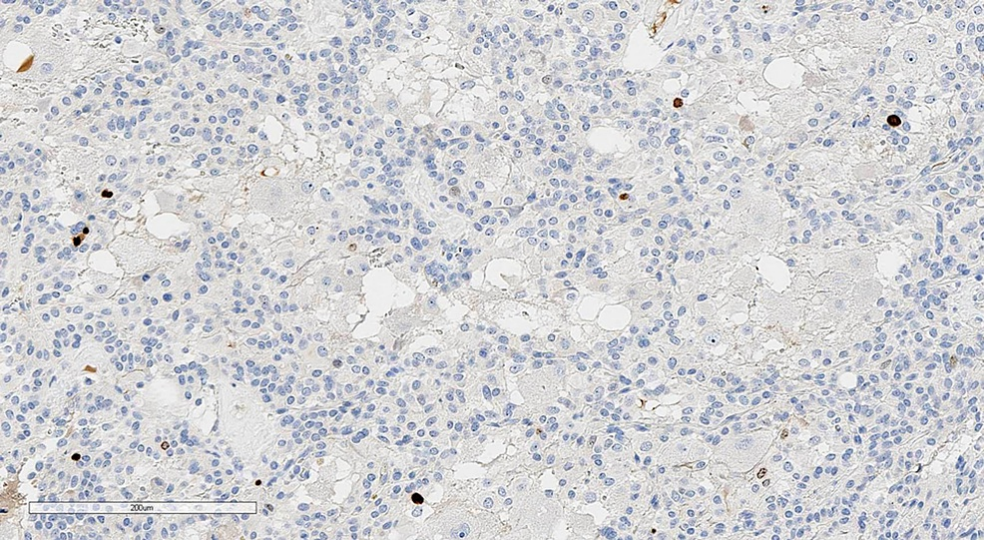
Immunohistochemical staining with Ki67 (×200): rare marks.

The pathological examination revealed that the tumor turned out to be trifractional, consisting of the following (Figures 7-10): monomorphic cells with oval nuclei - chief cells (type I), tending to perivascular arrangement, with sparse, inconspicuous supporting cells (type II); numerous clusters of large ganglion-like cells with rounded nuclei, distinct nucleoli, and abundant weakly granular cytoplasm; and a few schwannoma-like areas represented by a spindle cell component. Immunohistochemical study revealed immunopositivity of chief cells to synaptophysin and CKAE1/3, ganglion-like cells to synaptophysin, supporting cells to S100, and schwannoma-like regions to GFAP and S100. Thus, the morphological picture and immunophenotype of the tumor corresponded to gangliocytic paraganglioma.

## Discussion

Gangliocytic paraganglioma is a rare tumor with a good prognosis, usually arising in the small intestine (especially the duodenum) [2]. In a study by Okubo et al. in 2018, data from 263 patients with GP were collected and analyzed [2]. The vast majority of GP was detected in the duodenum (89.7%), and GP of the conus medullaris and cauda equina accounted for only 2.3% (six cases) [2].

In this regard, being an extremely rare tumor, GP remains poorly studied. In their study, Shankar et al. tried to explain how three types of cells (epithelioid, spindle-shaped (Schwann-like), and ganglion-like) can occur in a single tumor and noted that the combination of cytokeratin-positive neuroendocrine, ganglion, and Schwann cells suggests that all these cell types are of neoplastic origin [3]. On this basis, they concluded that this lesion reflects the ability of neural crest-derived sympathoadrenal cells to undergo divergent differentiation, leading to the formation of neuroendocrine and ganglionic cellular components. In addition, some studies have shown that hypoxia promotes the dedifferentiation of neuroblastic elements into immature neural crest cells [12].

Okubo et al. collected all available reports of GP and analyze treatment results [2]. It is worth noting that the number of studies analyzed by Okubo et al. appeared to be quite limited, and they seemed to describe tumors of duodenal origin. Therefore, this should be made clear in the discussion. They described GP as a tumor with an extremely good prognosis and only one recurrence, most likely associated with possible subtotal tumor resection. They argued that patients, after total tumor removal, did not require adjuvant therapy since there was no recurrence or metastatic spread. On the contrary, it is still unclear whether residual tumors of this type can be treated by radiation or chemotherapy alone without surgery [2].

It is worth emphasizing that for the patient, three months after surgery, no tumor recurrence was noted on the postoperative control MRI. We summarized the available data on surgical treatment of GP in Table 1.

**Table 1:** Present articles on the surgical treatment of GP at the cauda equina.

Author/year	Sex/age	Localization/size	Symptoms	Surgical approach	Results
Lerman et al. (1972) [1]	Male/29	Tumor at the level of L2-L1/2 × 2 cm	Lower back pain radiating to the left lower limb	Laminectomy L2-L3-L4	Total regression of symptoms
Schmitt et al. (1982) [4]	Male/33	Tumor at the level of L4-L5/4 × 2.5 × 2.5 cm	Lower back pain radiating to the left lower limb, paralysis of the elevating muscles of the left foot	Laminectomy L4-L5	Total regression of symptoms with a slight sensorimotor deficiency in the left leg
Djindjian et al. (1990) [5]	Male/36	Tumor expanded from just below the conus medullaris down to the L5 vertebrae/N/A	Lower back pain with lower paraplegia	Laminectomy L1-L5	Total regression of symptoms
Vural et al. (2008) [8]	Male/17	Tumor at the level of the L4, exiting through the right L4-L5 intervertebral foramen into the right psoas muscle/5 × 3 × 4.5 cm	Lower back pain with bilateral sciatica and difficulty in ambulation	Laminectomy L4 and right unilateral facetectomy	Total regression of symptoms
Shankar et al. (2010) [3]	Male/48	Tumor at the L2-L3 level/2.6 × 1.7 × 1.2 cm	Lower back pain and an intermittent tingling sensation in the inguinal area	Laminectomy L2-L3	Total regression of symptoms
Sable et al. (2014) [11]	Male/58	Tumor at the L2 level/2.5 × 2 × 1.5 cm	Lower back pain radiating to the right lower limb, mild weakness in the right external hallucis longus muscle (4/5)	N/A	Total regression of symptoms
Akbik et al. (2016) [7]	Male/68	Tumor at the L5-S2 level/6 × 6.2 cm	Perianal paresthesia and significant postvoid residuals	Laminectomy L5-S2	Marked improvement in paresthesia in the primary perianal distribution; however, daily catheterization for urinary retention was required
Nagose et al. (2019) [10]	Male/42	Tumor at the Th12-L2 level/3 × 2.5 × 2 cm	Difficulty walking, pain, and tingling in the right leg	Laminectomy	Total regression of symptoms
Lal et al. (2021) [9]	Male/35	Tumor at the Th11-L2 level/12 × 1.6 × 2.5 cm	Lower limb weakness	Laminectomy	Total regression of symptoms
Present case	Female/55	Tumor at the L4 level/1.35 × 1 cm	Lower back pain radiating to both lower limbs	L3-L4 interlaminar approach through a tubular retractor	Total regression of symptoms

Speaking about the choice of surgical approach, most recently, in their study, Lal et al. discussed the preference of laminotomy over laminectomy to prevent the formation of instability and other orthopedic problems at the level of the affected area of ​​the spinal column [9]. Helal et al. published a meta-analysis on minimally invasive surgery for extramedullary tumors of the spine. The authors concluded that minimally invasive spine surgery can be a favorable alternative with reduced blood loss, operative time, length of stay, and high rates of gross total resection [13]. Choi et al., in their paper, noted that both total and subtotal tumor resection can be achieved using a minimally invasive surgical approach with no significant difference in surgical outcomes [14].

## Conclusions

In our experience, patients with cauda equina root tumors, with the proper experience and skill of the surgeon, can and should be operated on using a minimally invasive approach (MISS) due to their best orthopedic postoperative outcome, which is no less important for patients.
